# Food fermentation in space: Opportunities and challenges

**DOI:** 10.1016/j.isci.2025.112189

**Published:** 2025-04-02

**Authors:** Maggie Coblentz, Joshua D. Evans, Caroline Isabel Kothe, Tiffany Mak, Nabila Rodríguez Valerón, Patrick Chwalek, Kim Wejendorp, Shilpa Garg, Louisa Pless, Sarah Mak, Pia M. Sörensen, Leonie Johanna Jahn, Ariel Ekblaw

**Affiliations:** 1 Space Exploration Initiative, MIT Media Lab, Massachusetts Institute of Technology, Cambridge, MA, USA; 2 Sustainable Food Innovation, The Novo Nordisk Foundation Center for Biosustainability, Technical University of Denmark, Kgs. Lyngby, Denmark; 3 School of Geography and the Environment, University of Oxford, Oxford, UK; 4 Microbial Foods, The Novo Nordisk Foundation Center for Biosustainability, Technical University of Denmark, Kgs. Lyngby, Denmark; 5 Basque Culinary Center, Facultad de Ciencias Gastronómicas, Mondragon Unibertsitatea, Donostia / San Sebastián, Gipuzkoa, Spain; 6 Responsive Environments, MIT Media Lab, Massachusetts Institute of Technology, Cambridge, MA, USA; 7 Sustainable Genomics Solutions, The Novo Nordisk Foundation Center for Biosustainability, Technical University of Denmark, Kgs. Lyngby, Denmark; 8 Center for Evolutionary Hologenomics, GLOBE Institute, University of Copenhagen, Copenhagen, Denmark; 9 Harvard John A. Paulson School of Engineering and Applied Sciences, Harvard University, Cambridge, MA, USA

**Keywords:** food biotechnology, microgravity sciences, food component analysis, food microbiology

## Abstract

Space exploration is expanding, which demands new technologies and enables new scientific questions. Food, as a bridge between disciplines, can bring these fundamental and applied goals together. Here, we investigate whether food fermentation in space is possible and if so, how it compares with fermentation on Earth. We fermented a miso, a traditional Japanese condiment, on the International Space Station over 30 days and compared it with two earthbound controls. Based on environmental metadata, shotgun metagenomics, whole-genome sequencing, untargeted metabolomics, colorimetry, and sensory analysis, we found that overall, the space miso is recognizable as a miso, indicating fermentation in space is possible. We also found some key microbiological and sensory differences in the space miso, which suggest distinctive features of the space environment. These findings can be harnessed to create more flavorful, nourishing foods for long-term space missions and invite further research questions across science, health, technology, and society.

## Highlights

• We fermented a miso on board the International Space Station
• Fermentation in space is possible with safe, successful results
• The space environment also shapes fermentation differently: a “space terroir”
• We lay a foundation for further interdisciplinary work on fermentation and space

## Graphical abstract

**Figure:**
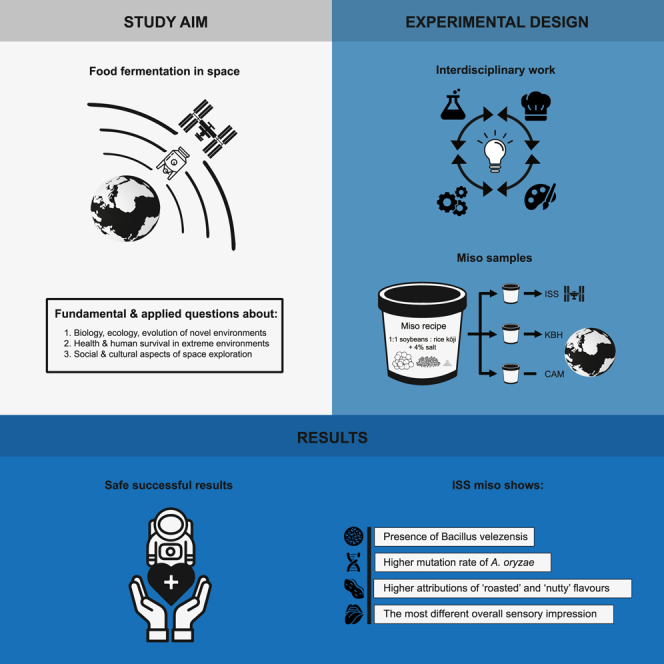

## Introduction

### Background

The International Space Station (ISS) is a laboratory and temporary home hosting a rotating crew of six international astronauts to conduct research for future space exploration. It orbits the Earth at a height of about 400 km, through what is known as Low Earth Orbit (LEO). It offers a unique environment for answering fundamental and applied questions, due to naturally occurring factors such as microgravity^1^ and increased radiation exposure^2^ and socio-technical factors including limited supply chain, isolation, and the imperative to form and reform a strong team across cultural differences. Food design and provisioning is one of the key challenges for sustaining life on the ISS and brings together these fundamental and applied questions.

The socio-technical complexity of space exploration puts significant constraints on food design. Safety, nutritional efficacy, weight, shelf-life, and cost are the main considerations. Entire departments are devoted to space food design and production: NASA’s Advanced Food Technology Project, for example, works on food for long duration missions such as a journey to Mars.^3^ Currently in the ISS, the highly engineered foods are mostly freeze-dried and reconstituted with recycled potable-quality water.^4^ Food and packaging waste is a great concern for the remote station where supply chain is limited.^5^ Aside from this preserved food, edible plants are being grown in the ISS with a Vegetable Production System called Veggie.^6^ In 2015, “Outredgeous red” Romaine became the first fresh food grown in the ISS to make its way onto the space menu.^7^ Yet plant growth time and astronaut labor make these experiments highly resource-consuming.^8^ Additional technical challenges for eating and preparing food in zero gravity environments include food “fly-aways” (when food floats away from the eater) and limited cooking equipment. A further socio-technical challenge is providing a broad enough range of food to accommodate the dietary and cultural needs of the six international astronauts the ISS hosts at a given time.

The complexity of space exploration also poses challenges to studying food in space. Sending biological materials to space for scientific experiments is costly and logistically challenging due to safety, storage, and transportation requirements. Studying fresh food samples in space is difficult since safety regulations restrict testing on human subjects (i.e., via consumption); most food needs to be tested on Earth first. There are also few data about sensory perception of food in space because historically the topic has not been prioritized by space agencies. Astronauts themselves have reported that in space their sense of taste and smell is reduced and that they prefer salty, spicy, and umami-rich foods.^9^^,^^10^

Fermentation can help address these technical, nutritional, and sensory challenges of space food design. Fermented foods are “foods made through desired microbial growth and enzymatic conversions of food components.”^11^ Recent decades have seen a revival of interest in fermentation traditions in many places in the world.^12^ This fermentation renaissance has been part of a growing interest in sustainable food practices and systems involving local production and the regeneration of biocultural diversity,^13^^,^^14^ and the rise of microbiome sciences revealing the importance of microbes to personal and planetary health.^15^^,^^16^

Although food fermentation has been practiced around the world for millennia,^17^^,^^18^^,^^19^ it has not yet entered the space environment. Since the fermentation process is shaped by its environment,^20^ new practices, flavors, and microbial communities may emerge as fermented foods migrate to outer space. This new research direction builds on the recent explosion of scientific interest in studying the microbiome of the ISS^21^^,^^22^^,^^23^^,^^24^^,^^25^ and the human microbiome in space.^26^ The ISS is a novel built environment in a larger non-terrestrial environment, but it is not hermetically sealed off from Earth nor is it in any way “sterile.” Astronauts from all over the world bring their microbes up to the ISS, which now has a distinct microbiome of its own that changes over time and shapes astronauts’ own microbiomes in turn.^27^ For these reasons, the ISS has become a rich site for microbiome research, both for health purposes and to learn about the fundamental dynamics of how microbiomes form and assemble in novel built environments.

Studying fermentation in space adds a new dimension to this research program, expanding interactions between human bodies and the built environment to include foods as a site of microbial exchange and testing the robustness of fermentation in novel extreme environments. The health benefits of fermented foods might also support astronaut health on future space missions. Though a few fermented and pasteurized products, such as kimchi and wine, have been sent to the ISS,^28^^,^^29^ and some fermentations have been modeled on Earth in “space-like” and “near-space” conditions,^30^^,^^31^ no actual process of food fermentation seems yet to have been carried out in space. The earlier spaceflights for already fermented and pasteurized products seemed to have nationalistic or commercial motivations, to promote cultural identity^28^ or increase a commodity’s market value; a kind of “space fetishism.”^29^ Here, we are interested in something else: using the uniqueness of the space environment to answer fundamental scientific and applied technical questions and to pose larger social questions relevant to space exploration and to life on Earth.

Therefore, in early March 2020, we sent a small container of miso-to-be up to the ISS. It stayed on board for 30 days to ferment, before returning to Earth as miso. With this experiment we had three main purposes: (1) to test the feasibility and robustness of fermentation in space; (2) to study how the space environment might shape microbial ecology, evolution, metabolism, and flavor in fermentation; and (3) to open up new multidisciplinary research directions across fundamental, applied, and social sciences. Although the experiment offers some new insights into miso, fermentation, and the space environment, the paper’s main contribution is methodological: to suggest how space fermentation can bring together fundamental science, health science, systems design, and social and cultural engagement in potentially groundbreaking ways.

### Experimental design

**Figure 1:**
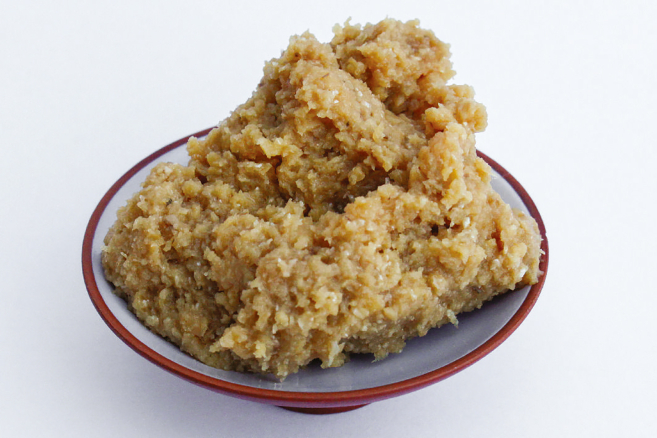
Miso appearance (Credit: J.D.E.).

Miso is a fermented, umami-rich paste from Japan, usually made from cooked soybeans, kōji (rice or barley fermented with the filamentous fungus *Aspergillus oryzae*), and salt (Figure 1 ^32^). This food product was selected for this experiment for several reasons. The first is practical: its firm, solid structure meant there was a reduced risk of leakage that could potentially damage other experiments and equipment on the ISS, and the timeframe for a young miso fit the 30-day window we had for the experiment. The second is scientific: this experiment fits within a recent surge of interest in and research on miso in the scientific community,^33^^,^^34^^,^^32^^,^^35^ which allows for comparison and contextualization of our results. This work is beginning to show the diversity and uniqueness of miso microbial communities, which our study builds on. The third reason is sensory: miso is a strongly flavored, umami-rich product that can satisfy astronauts’ need for flavor—its salty and pungent experience can enliven the senses in the sensory-muting environment of microgravity, which also has implications for astronaut diet and health.^10^^,^^36^ The fourth reason is health-related: miso is highly nutritious^37^ and has multiple health benefits that could serve astronauts.^33^^,^^38^^,^^39^ The fifth reason is cultural: fermentation is a ubiquitous and ancient cooking technique that everyone has some relationship to, knowingly or not. Miso, a fermented food from East Asia, was selected to diversify cultural and culinary representation in space.

**Figure 2:**
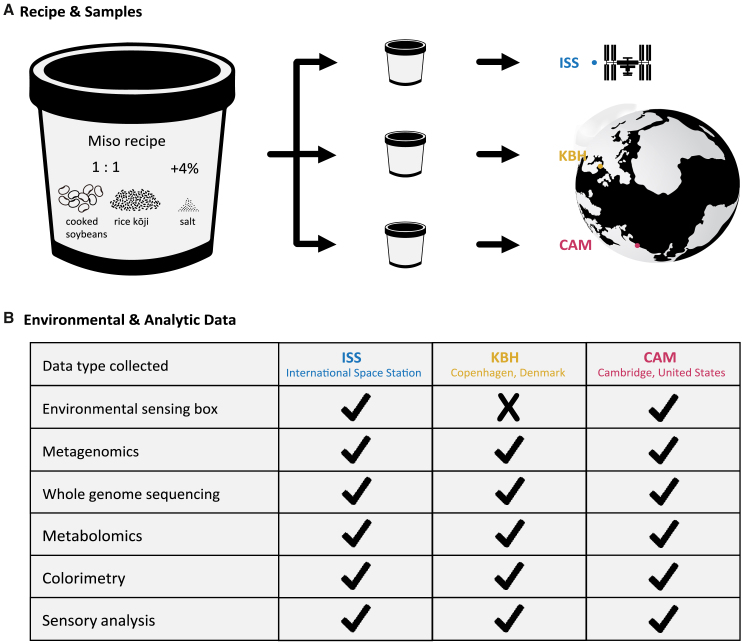
Schematic representation of the experimental design (A) Schematic representation of the recipe, sample division, and different locations where the misos fermented. (B) Summary table for the environmental and analytic data collected for each sample. “ISS” stands for the International Space Station, “KBH” for Copenhagen, Denmark, and “CAM” for Cambridge, Massachusetts, United States.

The miso mixture was prepared using cooked soybeans, rice kōji, and salt. A high-kōji low-salt style of young miso (Figure 2A) was prepared to facilitate a faster fermentation appropriate for the 30-day period. The miso mixture was produced in Copenhagen, Denmark in a food-safe environment according to a HACCP plan,^32^^,^^35^ split into three portions and kept frozen until the start of the experiment. The three misos were packed into identical plastic containers under a flow hood and fermented on the ISS, in Cambridge, Massachusetts, USA, and in Copenhagen, Denmark (Figure 2A). While on the ISS, the space miso was contained in an environmental sensing box, which measured temperature, relative humidity, pressure, off-gassing, light, and radiation (Figure 2B). The Cambridge miso (CAM) was also kept in an identical sensing box. The Copenhagen (KBH) miso was not, as only two sensing boxes were built, but was kept in a cupboard of similar dimensions. This difference gave us the opportunity to see how the sensing box itself might impact the miso’s fermenting environment. Temperature and relative humidity for the Copenhagen miso were manually recorded daily. Following the fermentation of the misos for 30 days, various analyses were performed (Figure 2B). Further details about the production, fermentation, and analysis of the miso can be found in the STAR Methods.

Sending experiments to space is costly, and space is at a premium. The little room we had we opted to fill with one maximal sample rather than three smaller ones, prioritizing successful fermentation over statistical robustness. This decision maximized the chance of the fermentation happening properly, though limits our ability to make conclusive claims about the similarities and differences among the misos, as we have no replicates to control for variation within the misos. We thus present our findings on the similarities and differences among the misos as exploratory, pointing the way for further studies.

## Results and discussion

### The space of space

The ISS is a unique space. Orbiting in LEO, it has distinct environmental conditions to those found on Earth. Two that are of particular relevance to this experiment are microgravity and increased radiation.^40^ The microgravity in LEO means that miso cannot be weighed down as it usually would, which might change how gas bubbles form and are released and the miso’s resulting density, how much oxygen is available, and how the microbial communities assemble and grow.^41^^,^^42^^,^^43^ Being outside Earth’s atmosphere also means the ISS is not as shielded from cosmic and solar radiation, which may also shape the microbial ecology and could potentially lead to higher mutation rates.^44^^,^^45^

**Figure 3:**
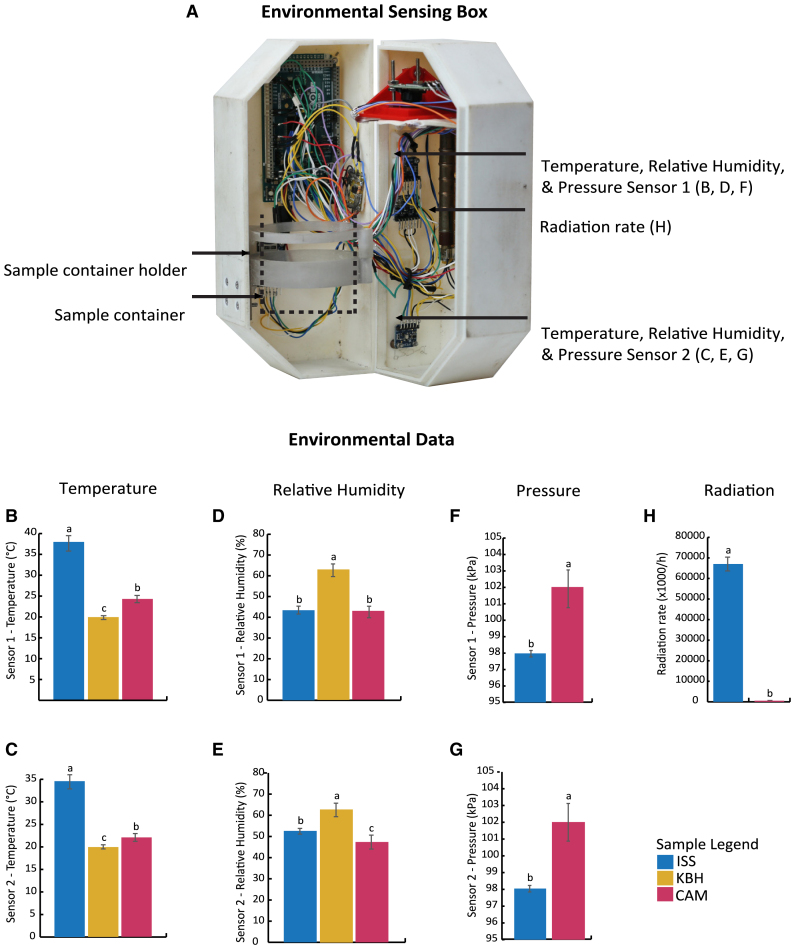
The environmental sensing box and its data (A) Labeled photo of the sensing box used to capture environmental data. (B–G) Bar plots representing mean measurements with standard deviation from Sensors 1 and 2 for temperature (B and C), relative humidity (D and E), and pressure (F and G). (H) Plot for radiation rate calculated from radiation measured separately with a Geiger counter. Significant differences are represented with letters. Full data can be found in Table S1.

To investigate the fermenting environment on the ISS and how it compared to the environments for the earthbound controls, the environmental sensing boxes collected metadata for temperature, relative humidity, pressure, and radiation (Figure 3). These data highlight the similarities and differences in environmental conditions between ISS and CAM. The manual temperature and relative humidity measurements for the KBH miso are also included in the graphs.

All temperatures presented statistical differences between the sites of fermentation (ANOVA, *p* < 0.05): on average (mean ± standard deviation [SD]), 36.3 ± 1.8°C for ISS, 23.1 ± 0.9°C for CAM, and 19.9 ± 0.5°C for KBH (for full data see Table S1.1). The higher temperature on the ISS may have been due to the sensing box being stowed tightly among other heat-generating equipment, raising the ambient temperature. The slightly higher temperature of the CAM miso supports the possibility that the electronics and enclosure of the sensing box generated and retained heat, raising the ambient temperature, as room temperature for the CAM sensing box, as for the KBH miso, was 20°. These differences in temperature are important, as they would likely affect microbial community formation and metabolism and sensory properties.

For relative humidity, there was a difference between the sensors: for Sensor 1, there was no statistical difference (ANOVA, *p* > 0.05) between sites (mean ± SD of 43.4 ± 1.9% for ISS, 42.8 ± 2.8% for CAM), whereas for Sensor 2, there was (mean ± SD: 52.6 ± 1.3% for ISS, 47.4 ± 3.2% for CAM, Tukey’s test, *p* < 0.05; for full data see Table S1.2). KBH had a mean relative humidity of 62.9 ± 3.1%. It is unclear why Sensor 1 should have recorded lower humidity than Sensor 2 at both sites. The higher relative humidity for KBH may simply have been because the ambient humidity in the room where the miso fermented was higher than in the CAM and ISS sensing boxes.

For pressure, the sensors recorded means of 98.0 ± 0.2 kPa for ISS and 102.0 ± 1.1 kPa for CAM, a statistically significant difference of 4.0 kPa (ANOVA, *p* < 0.05; for full data see Table S1.3). This difference was surprising, as air pressure on the ISS is supposedly held at 101.3 kPa, the same as sea level on Earth.^46^ Nonetheless, it is unlikely that this discrepancy is due to sensor malfunction, as both pressure sensors recorded the same results.

For radiation, the radiation rate on the ISS (mean of 67024 clicks/h) was 120 times higher than on Earth (mean of 559 clicks/h; for full data see Table S1.4).

### Similarities and differences among the misos

Our analysis of the microbial communities, flavor compounds, and sensory properties of the misos show that (1) overall, the space miso is a miso, and (2) there seem to be differences between the misos that suggest a specific fermentation environment in space. For analysis, we took samples from three portions—the top, middle, and bottom of the jars—to investigate how the microbial communities and flavor compounds might also differ within each miso. Any apparent similarities and differences among the misos and their portions would require experimental replicates to confirm. We therefore present them here tentatively and as suggestions for further research.

#### Microbial communities

In analyzing the microbial communities of the misos, we investigated their taxonomic composition, the mutation of *Aspergillus oryzae*, and safety.

##### Taxonomic composition

We mapped the metagenomic reads against a genomic database (MetaPhlAn) and identified between 11 and 15 bacterial species per miso (18 in total across all samples) and *A. oryzae* as the only eukaryotic species, found in all samples (Table S2.1). To characterize the microbiota independently of the set reference genomes, we also conducted an analysis using the leuS marker gene (NCBI protein database) from the assembled metagenomes. This analysis yielded between 7 and 10 bacterial species per miso (14 in total across all samples) and *A. oryzae* as the only eukaryote (Table S2.2). For comparison, the only other study of miso ecology using metagenomic sequencing found between 19 and 48 species in six novel misos using MetaPhlAn and between 4 and 11 species using the leuS marker gene.^32^ So these misos are on the lower end of species richness for MetaPhlAn mapping and with comparable species richness for leuS marker gene analysis. The absence of non-*Aspergillus* eukaryotes is surprising, as salt-tolerant yeasts are a common feature of miso ecology.

The low richness for these misos could have been due to the relatively sanitized mode of production compared with traditional miso—ingredients were processed with gloved hands, mixed and packed into sterilized containers, and transferred only under flow hood. We did this to try to limit confounds of microbes from the producer’s body, locations of production, or sampling from influencing the ecology, to isolate the effect of the space environment. This meant there were no opportunities for microbes to enter the miso after it was made. It could also have been due to the freezing of the mixture after preparation and before fermentation, which would have killed some of the taxa in the mixture before they had a chance to grow. Freezing before fermentation is not part of traditional miso making, and this could explain the absence in these misos of many of the typical miso-associated taxa from the literature.

**Figure 4:**
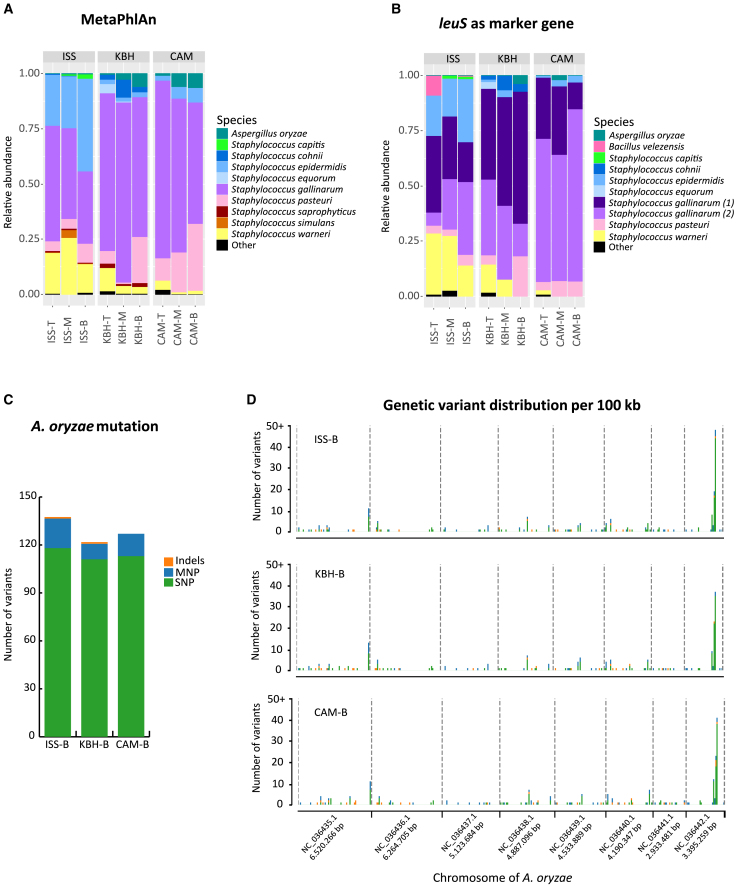
Microbial composition of ISS, KBH, and CAM misos, and mutation rates and genomic distribution of variants for *A. oryzae* isolated from each (A and B) Bar plots representing the relative abundance of microbial communities in misos fermented in the International Space Station (ISS); Copenhagen, Denmark (KBH); and Cambridge, Massachusetts, USA (CAM). The sampled portions are denoted by T (top), M (middle), and B (bottom). The relative abundances in microbial composition were calculated from the reads using the MetaPhlAn database (A) and from the coverage of the leuS marker gene assembled from the metagenomes (B). (C) Bar plots representing the mutation rates of *A. oryzae* isolated from the bottom portion of each miso. The rates of mutation were determined by comparison with the sequenced genome of *A. oryzae* isolated from kōji grown using the same spore as that used to make the kōji for the ISS, KBH, and CAM misos. Genetic variants were characterized by a combination of single-nucleotide polymorphisms (SNP) represented in green, multi-nucleotide polymorphisms (MNP) represented in blue, and insertion-deletion mutations (Indels) represented in orange. (D) Multi-stacked plots representing distribution of genetic variants across the genome for *A. oryzae* isolated from the bottom portion of each miso. Each chromosome is divided into 100 kb segments to display the number of variants of each type. Full data can be found in Table S2.

Some similarities emerged across the samples. Although the *A. oryzae* from the kōji was identified in all samples, only small proportions were detected and mainly in the ISS samples (Figure 4A). This relative absence may be due to a bias in DNA extraction methods that more easily break down prokaryotic cells.^47^ Even methods optimized for fungal extraction have been known to extract less of *Aspergillus* spp.^48^ The bottom portions of all three misos had the highest relative abundance of *A. oryzae*. We would expect to find *A. oryzae*, a strict aerobe, mostly at the top of the miso. However, this finding may rather be due to lower overall microbial abundance at the bottom of the miso, which would make the *A. oryzae* DNA seem more prominent. Among the bacterial species identified, the majority belong to the genus *Staphylococcus*. The *Staphylococcus* spp. detected in all misos belong to *S. gallinarum* (two different subspecies/strains), *S. epidermidis*, *S. pasteuri*, and *S. warneri* (Figure 4B).

There is not so much literature on the microbial communities of miso (at least in English) to help contextualize our findings. What does exist is mostly based on culture-dependent techniques that cannot detect the communities’ full diversity,^33^ and the only studies that use culture-independent techniques have focused on novel misos (misos made with untraditional substrates, like yellow peas or lentils), not traditional ones.^32^^,^^35^ A recent review of miso microbial communities shows that multiple *Staphylococcus* spp. (*S. gallinarum* and *S. kloosii*) are commonly found in miso.^33^ Our misos suggest further *Staphylococcus* species might be added to this list, a finding supported by the recent culture-independent studies of novel misos,^32^^,^^35^ one of which also shows evident adaptation of human-skin-associated *S. epidermidis* to the miso niche.^35^ Other bacterial taxa often found in miso include *Bacillus* spp., *Enterococcus* spp., Lactobacillales, *Tetragenococcus* spp., and *Weissella* spp. None of these were found in our misos in significant amounts, which could be related to the relatively “clean” way they were produced and/or the freezing mentioned above.

Overall, we can observe that each miso presented a similar microbial composition throughout its top, middle, and bottom portions. There thus seems to be more variation among sample locations than among positions in the miso. Although this is not possible to confirm statistically as we only have one miso per location, it is visually suggested in the bar plots (Figures 4A and 4B). Within the overall finding of the space miso being a recognizable miso comparable to the earthbound ones, this observation of potential differences among the samples leads us to consider the space miso’s apparent specificities.

In comparison to the Earth misos (KBH and CAM), the ISS miso presents a higher proportion of *S. epidermidis* and *S. warneri*. These species may have been favored by the higher temperatures on the ISS (36.3°C). Furthermore, one species, *Bacillus velezensis*, was only detected in the ISS samples and mainly in the top (Figure 4B). This species has been previously isolated from fermented soy foods such as meju and doenjang^49^^,^^50^^,^^51^ and is listed as safe in food by multiple national and supranational food safety authorities.^52^^,^^53^ *B. velezensis* is an aerobe, and many strains have an optimal growth temperature of 30°C–40°C,^54^^,^^55^^,^^56^ which could explain its presence in only the top portion of the ISS miso.

Studies have detected *B. amyloliquefaciens*,^32^^,^^35^^,^^57^ a closely related species to *B. velezensis*,^58^ in a variety of traditional misos and novel ones made with alternative plant substrates, which support the presence of multiple *Bacillus* spp. in miso.^33^ Other studies indicate that *Bacillus amyloliquefaciens* is used in the production and processing of kōji, the essential ingredient in miso production.^59^ This suggests that besides *A. oryzae*, certain *Bacillus* strains may be part of some spore starter cultures and may have entered into the misos this way. Another explanation for their presence in miso is that they occur as seed endophytes in pulses and their heat-resistant spores are not inactivated by cooking, allowing them to enter into the miso via the leguminous substrate.

##### *A. oryzae* mutation

To explore whether the space environment might also shape microbial evolution, we calculated the mutation rate of *A. oryzae* in the different environments. *A. oryzae* was selected because we knew it was in each miso and that it would grow on plates. We chose to isolate it from the bottom portion of each miso, because that portion contained the highest relative abundance of *A. oryzae* DNA, and from each portion of the space miso. One sample of *A. oryzae* was isolated from each of these five portions, and genomic analysis was performed to compare the samples to the reference strain grown from the same spore.

All samples presented more than 120 variants compared to the control reference strain, which were characterized by a combination of single- and multi-nucleotide polymorphisms (SNP and MNP, respectively) and insertion-deletion mutations (Indels; Figure 4C). These variants are potentially a result of the fermentation process, as suggested by some studies of *Saccharomyces cerevisiae* in wine fermentation, where the genetic diversity of some strains has been shown to change in response to stresses imposed during fermentation.^60^ Notably, the number of variants is highest in the ISS miso (Figure 4C), in all portions (Table S2.3), which could have been driven by the harsher space environment, especially increased exposure to radiation (Figure 3H) for which there is precedent in fungi.^45^ To confirm this finding, triplicates should also be isolated from the KBH and CAM misos to ensure valid comparison and statistical significance.

An analysis of the distribution of these variants across the genome was also performed, revealing a clustering of variants in the eighth chromosome across all samples, and particularly in the ISS sample (Figure 4D). This finding is validated by the *A. oryzae* genomes isolated from the other two portions of the space miso (T and M; see Table S2.3). Possible explanations include that this chromosome is particularly vulnerable to damage and/or mutation, and/or that it has lower efficiency in DNA repair mechanisms than the other chromosomes. Functional genomic analysis could offer insight into this question.

##### Safety

As one of the key aims for exploring fermentation in space is to determine a product’s consumability, it was important to investigate the misos’ safety based on microbial species composition—here in particular with regard to the *Staphylococcus* spp. present in all the misos.

The most abundant *Staphylococcus* species detected in the misos are coagulase-negative Staphylococci (CoNS), which are occasionally found in fermented foods worldwide^61^ and may be part of normal human skin flora.^62^ Many CoNS are used as starters for cheese and meat fermentation to enhance color and flavor development^63^^,^^64^^,^^65^^,^^66^^,^^67^ and have been investigated for use as starters in soybean fermentation.^68^

Some CoNS may carry enterotoxin genes such as pyrogenic toxin superantigen (PTSAg) and exfoliative toxins.^62^ We searched for the presence of genes encoding for PTSAgs (sea-see, seg-sevu, selv, selx, sey, selz, sel26, sel27, and TSST1) and exfoliative toxins (eta, etb, etd). We detected only one virulence gene (sel26) in the top portion of the ISS sample, which corresponds to *S. aureus* present in low abundance in this sample (Table S2.4). It is important to highlight that the top portions of miso are usually removed and discarded before the rest of the miso is eaten, as we did here. This finding may be supportive of this practice. The other Staphylococci detected in higher abundance are not of food safety concern. No pathogenic species were detected in the other portions or samples. These provisional findings based on the metagenomic data suggest that the space miso is safe to eat. This conclusion would require culture-dependent methods to confirm, as some pathogens can pose risk even at levels lower than the detection threshold for sequencing. There is effectively no tolerance for the risk of food-borne illness in space,^69^ so further tests would be required to ensure safety for space-based fermentation.

#### Flavor

To further investigate the similarities and differences between the space and Earth misos as foods, and ultimately determine whether the space miso was indeed a miso, we also investigated the misos’ flavor chemistry and sensory qualities. For volatile aroma compounds and amino and organic acids, we analyzed all portions of the misos (top, middle, and bottom), to characterize the miso as a complete system. For sensory analysis, we removed and discarded the top portion and combined the middle and bottom portions, as would be done when preparing miso for consumption.

##### Volatile aroma compounds

**Figure 5:**
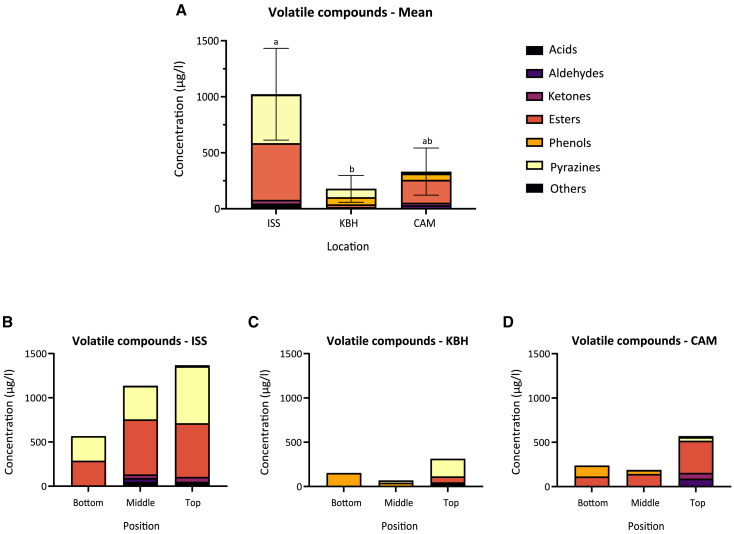
Concentrations of volatile compounds (A–D) Stacked bar plots of volatile compounds grouped according to compound class for all misos averaged (mean) across all portions of the miso jar (top, middle, and bottom; A) and for individual portions within ISS (B), KBH (C), and CAM (D). Error bars in (A) represent the SD values of the averaged concentrations across portions of each miso. Significant differences are represented with letters. Full data can be found in Table S3.1.

Most of the compound classes (six out of seven) were present in all the misos (Figure 5), suggesting an overall aromatic comparability between the space miso and Earth misos. Previous studies of miso detected aldehydes, ketones, esters, and pyrazines as the main compound classes in miso,^70^ all of which are present here (Figure 5A), suggesting that these misos are also comparable to traditional ones. A more quantitative comparison with traditional misos may be further illuminating, though would require careful alignment of methodology to ensure validity.

Most compound classes are present across all the misos (Figure 5A). The mean concentration of aroma compounds is significantly higher in the ISS miso (1020 ± 410 μg/L) than in KBH (177 ± 120 μg/L; it is also higher than in CAM, 332 ± 210 μg/L, though not significantly; Kruskal-Wallis, *p* < 0.05; Table S3.1). The concentration of aroma compounds seems to be positively correlated with the fermentation temperature (Figures 3B and 3C). In particular, the ISS miso contains significantly higher concentrations of esters and pyrazines—22.6 times as much esters and 5.8 times as much pyrazines in ISS than in KBH and 29.9 times as much pyrazines in ISS than in CAM (Kruskal-Wallis, *p* < 0.05; Table S3.1). Pyrazines are formed by the Maillard reaction between amino acids and reducing sugars,^71^ a reaction accelerated by heat. The higher temperature of the ISS miso could therefore have been responsible for the ISS miso’s higher levels of pyrazines.

The ISS miso also contains by far the highest concentration of the main ester found across most samples, the honey-like phenylacetic acid methyl ester—510 ± 190 μg/L, compared with 19 ± 32 μg/L in KBH and 190 ± 120 μg/L in CAM (Table S3.1). The difference between ISS and KBH is significant but not that between ISS and CAM or between KBH and CAM (Kruskal-Wallis, *p* < 0.05; Table S3.1). The one aromatic acid detected in the misos, 2-methyl-butanoic acid, has a cheesy aroma.^72^^,^^73^ It was found in the top and middle portions of the ISS miso, to a lesser degree in the top portion of KBH, and not in any of the other portions (Table S3.1) and correlates with the relative cheesiness of the misos from the sensory analysis (Figure 7A).

Certain patterns also appear in the portions of the miso across the different locations. In general, the top portions have more aroma compounds than the middle and bottom portions. This may be due to different mechanisms for pyrazines and esters, respectively, the two most abundant compound classes. It is possible that the top of the miso, exposed to the ambient heat in the air, could have had higher rates of Maillard reaction, yielding more pyrazines. We do not have temperature measurements of the miso surface to say for certain. Esters, meanwhile, can be formed through reactions between carboxylic acids and alcohols. These esterification reactions also increase with temperature.^74^ The presence of oxygen facilitates the oxidation of alcohols to aldehydes, which can further react with carboxylic acids to form esters. Thus the presence of oxygen could also have indirectly encouraged ester formation in the top portion.^75^ Meanwhile, the one phenolic compound—2-methoxy-4-vinylphenol, having a spicy, clove-like, roasted peanut aroma, common in buckwheat^76^—is found only in the middle and bottom portions of KBH and CAM and not in ISS. Its formation may have been favored by the cooler temperatures and anoxic conditions of these miso portions.

##### Free amino and organic acids

**Figure 6:**
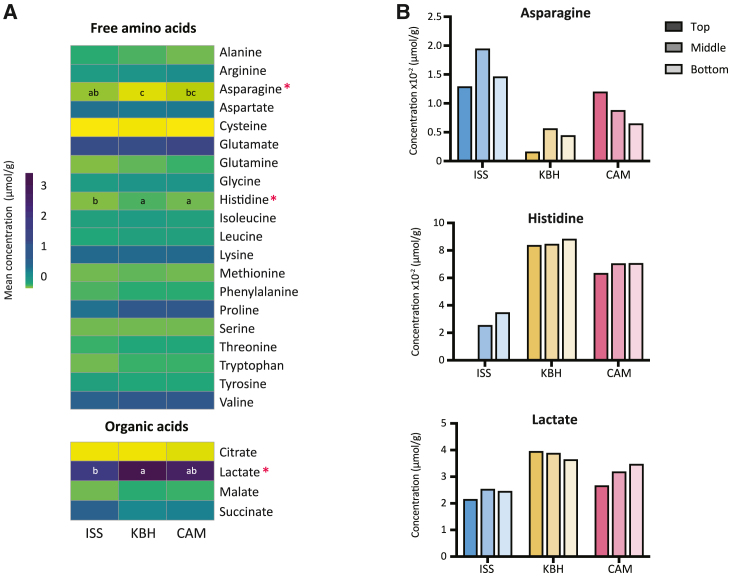
Free amino and organic acid compositions (A) Heatmap of all free amino and organic acids detected in the miso samples (means of top, middle, and bottom portions); acids with statistically significant differences between sample means are marked with an asterisk. Significant differences are represented with letters. (B) Bar plots of relative concentrations of acids with statistically significant differences: asparagine, histidine, and lactate. Full data can be found in Table S3.2.

We analyzed free amino and organic acids to understand their contribution to the basic taste sensations, in particular those arising from microbial metabolism in miso: umami and sourness. Overall the free amino acid profile is similar among all misos (Figure 6A; Table S3.2). The most abundant free amino acid across all samples is glutamate, known for its characteristic umami taste, which is hydrolyzed by glutaminase from glutamine liberated from soy proteins in the fermentation process.^77^ Glutamate and aspartate have been found to be the most abundant free amino acids in different misos before, so our findings are in accordance with the literature.^78^

There are three acids with statistically significant differences between some of the misos (Kruskal-Wallis, *p* < 0.05): the amino acids histidine and asparagine and the organic acid lactate (Figure 6). Lactate is a compound commonly found in many fermented foods, often produced by lactic acid bacteria and contributing to foods’ organoleptic properties and stability.^79^^,^^80^ Here, it was likely produced by the *Staphylococcus* spp., some of which are also known to produce lactic acid.^81^ The KBH miso had the most lactate, followed by CAM and ISS.

Histidine is more abundant in the Earth misos. Histidine is 6% of soybeans’ total amino acid composition.^82^ In soy sauce production, histidine has been observed to increase in the early stages of the fermentation, then decrease over time.^83^ We might mainly see free histidine as a result of initial degradation of plant protein, which would then decrease after uptake by some of the microbial community in the misos. Given that the misos were all started from the same batch with the same kōji, the difference in histidine concentration between the space and earth samples could be due to the different rates of substrate breakdown and metabolic activity from temperature differences and other environmental conditions increasing the fermentation rate in space.

Asparagine, meanwhile, is more abundant in the ISS miso than in KBH and CAM. Asparagine is fairly abundant in soybeans, with 10% of the total amino acid concentration,^82^ and is unlikely to be taken up and catabolized by *A. oryzae* as it enters late in the tricarboxylic acid cycle.^83^ Consequently its higher abundance in the ISS miso could indicate higher proteolytic activity, i.e. a more aged miso.

##### Sensory analysis

Visual observations indicated that all of the misos had significant white mold growth on the surface. Usually one covers a miso during fermentation (with a wooden board, plastic cling film, or other material) to minimize its exposure to oxygen and inhibit mold growth on the surface. Because of the microgravity conditions on the ISS, it was not possible to cover the space miso as one would usually do. This white mold growth was likely the *A. oryzae*. The space miso also had much more of a thick brown liquid on top than the Earth misos. This viscous liquid was tamari, a naturally occurring product that occurs as moisture is freed from the plant substrates during the fermentation and rises to the top of the miso. The greater presence of tamari in the ISS miso was likely due to increased rate of fermentation from higher temperatures and more disturbance during travel. The ISS miso was also darker than the Earth misos. This may also have been due to the higher fermentation temperature and possibly higher oxidation from being jostled more during transport. This combination of white mold growth, tamari, and oxidation on the surface supported our decision, based on miso tradition, to remove the top layer before the sensory analysis.

**Figure 7:**
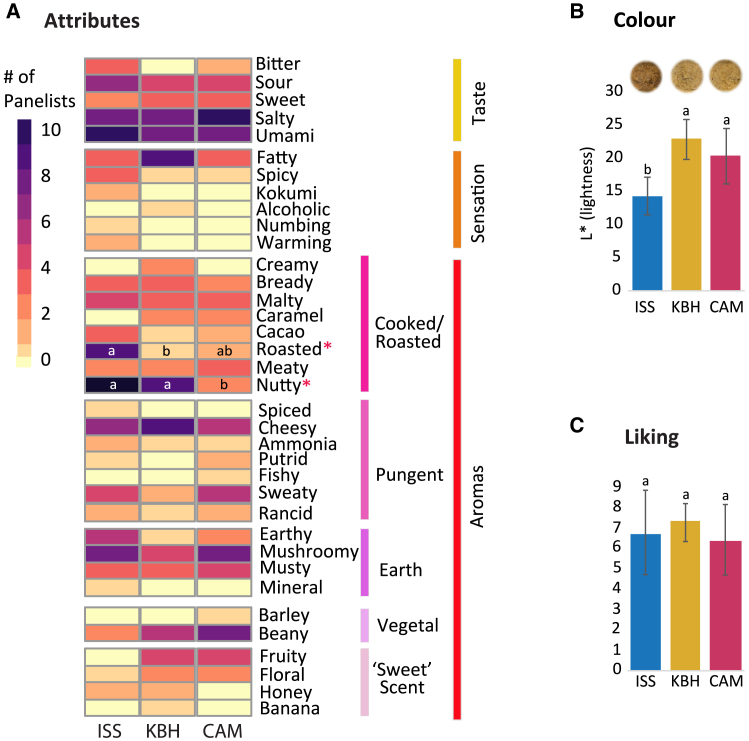
Sensory qualities of the misos (A) Sensory attributes reported by the panelists in the sensory analysis; attributes with statistically significant differences between the misos are marked with an asterisk. (B) Mean color of the misos using lightness variable L∗; error bars represent SD between portions, letters represent significant differences. (C) Overall acceptability (liking) of the misos using hedonic rating; error bars represent SD between panelists, letters represent significant differences. Full data can be found in Table S4.

We later measured this difference in color and found the space miso was indeed statistically darker than the Earth misos (Tukey’s test, *p* < 0.01; Figure 7B; Table S4.7). The space miso’s darker color is linked to the greater production of pyrazines (Figure 5), which can be explained by Maillard reactions and/or Strecker degradation; the latter occurs in the relative absence of reducing sugars.^84^ The formation of pyrazines has also been described in other longer-fermented foods, such as parmesan cheese, where browning is sometimes also observed.^84^ Pyrazines are reported to display baked, roasted, and nutty flavor characteristics.^85^^,^^86^ In our sensory analysis, these aromas were statistically perceived as more prevalent in the space miso (Cochran’s Q test, *p* < 0.05; Figure 7A; Table S4.5).

In the sensory analysis, overall the KBH miso was liked most (7.43 ± 2.08), followed by the ISS miso (6.79 ± 0.94) and the CAM miso (6.43 ± 1.74; Figure 7C; Table S4.4). All misos were liked more than neutral, and statistically, all misos were liked equally (ANOVA, *p* > 0.05). This indicates that the space miso was accepted as a miso.

The similarity among the misos is supported by certain sensory attributes. The main similarity in the taste is the high ratings compared to most other attributes for “umami” and “salty” across all samples.^87^^,^^88^^,^^89^ “Cheesy” and “mushroomy” aromas are also high compared to most other attributes in all samples (Figure 7A), both of which seem to be positive attributes contributing to overall liking.

From here, some interesting differences emerge. The ISS miso exhibits some clear sensory differences compared with the Earth misos. As mentioned, the ISS miso has statistically higher ratings for “roasted” and “nutty” aromas compared with the Earth misos (Cochran’s Q test, *p* < 0.05; Figure 7A). These attributes are associated with pyrazines and likely come from the increased temperature of the ISS miso, which would have sped up fermentation.^90^

Some of the samples also exhibit characteristic “off” flavors. KBH and especially CAM show a “beany” taste. This is generally considered an off-flavor and might help explain CAM’s lowest liking score. This beaniness is more likely to occur in underfermented misos, due to the soybeans not being sufficiently broken down, which here is correlated with the lower fermentation temperatures of KBH and CAM.

In the Projective Mapping test for total sensory difference between the misos, panelists rated ISS as 2.9 times more different from KBH and 2.6 times more different from CAM compared to the difference between the Earth misos (Table S4.6). This indicates that the ISS miso tasted most distinct of the three.

### Conclusions and future directions

Combining our metagenomic, metabolomic, and sensory data, we conclude that the space miso is a recognizable and safe miso. This finding suggests that other types of food fermentation might also be performed safely and successfully in space. Digging deeper into analysis and including our genomic and colorimetric data, we find some notable, though still provisional, differences among the misos, in microbial composition, mutation rate, flavor chemistry, color, and sensory profiles—not only between the space and Earth misos but also between the two Earth misos and in certain cases among the different portions of the miso. These differences are to some extent to be expected and are an extension of the more general pattern on Earth of how different conditions shape the microbes, metabolisms, and flavors of the same fermentation in different ways. In this case, the fermentation process seemed to go faster in space—likely due to the increased temperature in the sensing box on the ISS and possibly to the disturbance from travel and/or other factors.

The logistical factors that complicate any space experiments lead us to reflect on the challenges and limitations of our study. We have already reflected on our decision to prioritize sample size over replicates, favoring the likelihood of successful fermentation over the possibility of statistical comparison. Though we believe we have been able to demonstrate the former, the lack of the latter inhibits our ability to make conclusive claims about the similarities and differences among the misos. Furthermore, to send an experiment to space at all requires navigating a complex system of regulations and constraints that shapes how experiments can be planned and carried out. For example, transport logistics meant it was not possible for the miso mixture to stay frozen until on the ISS. It entered ambient temperature—at times possibly higher and lower than room temperature, due to heat generated by other equipment and/or cooler temperatures during shipping—when shipped from Cambridge, Massachusetts to mission control in Houston, Texas, then on to Florida for launch. And there passed some days between it leaving the ISS and being retrieved from the Pacific before being shipped back to Cambridge, when it could be returned to the freezer and the fermentation stopped. We controlled for this variability by keeping our earthbound misos in and out of the freezer for the same periods of time, and the time spent out of the freezer to a minimum (see Table S1.5 for the dates of the three misos’ respective journeys, and Figure S1 in Document S1 for aligned temperature curves for the misos before, during and after the experiment). The space miso of course also underwent more travel than the two controls, which stayed stationary throughout the experiment. This travel meant that the space miso received more physical disturbance, which may have affected its fermentation.

These logistical limitations inevitably shaped the experiment and findings. But understanding them as confounding factors is not quite possible because they are unavoidable parts of getting something to, holding something in, and retrieving something from space within current infrastructure. A different and perhaps more fruitful way to account for them is as part of space’s “terroir”—the system of natural and cultural features (e.g., location, soil, topography, climate, microbes, built environment, traditional practices, and knowledge) that shape food production in specific ways, yielding a “taste of place.”^91^ While some factors that shaped the experiment, like radiation and microgravity, are more natural features of space itself, others, like increased temperature and physical disturbance, are more cultural ones, part of the complex socio-technical systems that currently allow us access to it. This concept of “space terroir” thus offers multiple advantages. First, it invites us to notice the specific features of the space environment that shape fermentation processes in unique ways. Second, it helps us differentiate these features, avoiding mis-ascribing to the space environment, for example, differences that may have more to do with socio-technical systems design. And third, it opens up promising new directions for further research. In closing, we would like to outline some of them across four interrelated themes: fundamental science, health, systems design, and society and culture.

Fermentation in space can address fundamental questions in ecology and evolution, metabolism, flavor chemistry, and other scientific fields. We can use it to learn more about the biology of the space environment, how its features like radiation and microgravity shape microbial life and flavor formation, and how familiar microbes and microbial ecologies change as they migrate to unprecedented and novel environments. As we discuss here, further research in this vein with replicates will be able to investigate these patterns more definitively and correlate metagenomic, functional, and metabolomic datasets. The current popular interest in fermentation and the enduring fascination with space also make fermentation in space a promising way to engage multiple publics in science, which we have experienced here.^92^^,^^93^

Fermentation in space raises questions for health research—not only physical health and productivity but also mental and emotional health and well-being and their connections to sensory satiety, pleasure, and enjoyment. Fermentation in space can offer astronauts improved nourishment and gut health, which is linked to behavior and cognitive performance.^15^^,^^94^ Fermented foods may help alleviate sensory-specific satiety,^95^^,^^96^ or flavor boredom, which can arise from astronauts’ current predetermined diets and negatively impact their well-being and nutrient intake.^10^ Engaging astronauts in fermenting their own foods might increase their sense of agency and enjoyment in eating, and thus their well-being and overall performance, by letting them customize their food more, especially with the strong and pungent flavors they crave (ibid.). Further experiments might be designed to let astronauts taste products fermented on the ISS and study their response.

Fermentation in space can contribute to space exploration design and systems engineering. It offers techniques that could expand the foods available to astronauts, preserving fresh foods for longer than a few days after launch and restocking and offering nutrition and flavor using fewer resources than on-board plant cultivation. As a passive form of cooking that does not require much extra infrastructure, it could be used to upcycle food waste during spaceflight.^3^ For longer deep space missions, cargo resupply missions may not be possible, and it is unclear how food quality and nutrients will degrade in these extended conditions. In these conditions, it will become increasingly important for astronauts to be able to produce and preserve their own food, and fermentation can be a crucial tool here. Our proof of concept that fermentation is possible in space can support this future work, adapting other fermentation processes to the space environment and developing the systems of quality assurance to make it feasible.

Finally, emerging space fermentation practices suggest broader social and cultural questions. Fermentation in space might invite new forms of culinary expression, creating foods tailored to the different sensory environment of space, where sensory perception is altered. It could expand and diversify cultural representation in space, a new frontier for “gastro-diplomacy,”^97^ through the exchange and development of treasured foods and flavors. As such, it raises important questions about how we can increase and diversify access to both scientific research and space exploration, especially as a “new space age” of public space agencies and new private space start-ups continue to evolve.

### Limitations of the study

This study has a few limitations, including low number of samples, lack of replicates, and constraints associated with the logistics of space travel. We discuss these limitations, our approach to navigating them, our rationale for this approach, and how we account for them in the Conclusion.

## Resource availability

### Lead contact

Further information and requests for resources and reagents should be directed to and will be fulfilled by the lead contact, Joshua D. Evans (joshuae@dtu.dk).

### Materials availability

This study did not generate new unique reagents.

### Data and code availability

• Data: the raw sequences of the miso metagenomes and *A. oryzae* genomes have been deposited in the European Nucleotide Archive (ENA) and NCBI and are publicly available as of the date of publication. Processed genomic, metagenomic, and metabolomic data, including BAM and VCF files, assembled contigs, predicted genes, marker genes, and genes encoding for enterotoxins, have been deposited in Mendeley Data. Accession numbers are listed in the key resources table.
• Code: this paper does not report original code.
• Additional information: Any additional information required to reanalyze the data reported in this paper is available from the lead contact upon request.

## Acknowledgments

We would like to thank Lars Williams, Mark Emil Hermansen, Chris Stewart, Eric Heilig, and the team at Empirical Spirits, Copenhagen, for hosting and facilitating J.D.E. making the space miso; Peter Dilworth and Jamie Milliken at MIT Media Lab, for their mechanical and electrical engineering support for the environmental sensing box; Jennifer Wang and Charles Vidoudez at the Harvard Center for Mass Spectrometry for technical support with metabolomics analysis; Tom Gilbert at the University of Copenhagen for facilitating preliminary metabarcoding of the misos; Mariana Arrango Saavedra, Line Sondt-Marcussen, and Vijayalakshmi Kandasamy at the Center for Biosustainability at the Technical University of Denmark for support with the metagenomic sequencing of the samples; Morten Otto Alexander Sommer at the Center for Biosustainability at the Technical University of Denmark for supervisory support of T.M. and L.J.J.; and our diverse group of natural scientists, social scientists, chefs, fermenters, and designers who participated in the sensory analysis. The ISS experiment, preliminary metabarcoding, and metabolomics were funded by the MIT Media Lab Space Exploration Initiative. The metagenomics and genomics were funded by The Novo Nordisk Foundation, 10.13039/501100009708NNF Grant number NNF20CC0035580.

## Author contributions

J.D.E. and M.C. conceived the study and designed the experiment. M.C. prepared the experiment plan and cleared it through regulation. J.D.E. prepared the miso. P.C. supported the sensing box hardware and firmware design, integration into Nanoracks BlackBox, and preliminary environmental data analysis. L.P. conducted a preliminary DNA extraction, metabarcoding, and analysis, supervised by S.M. K.W. contributed to early discussions of what analyses to conduct and how to structure the paper. T.M., C.I.K., N.R.V., and L.J.J. helped plan and conducted metagenomics, genomics, metabolomics, colorimetry, and sensory data collection and analyses. P.S. supervised the generation and analysis of the metabolomics data. J.D.E., M.C., T.M., C.I.K., N.R.V., and L.J.J. iterated the analyses and outlined the article. J.D.E. wrote the first draft of the manuscript. J.D.E., M.C., T.M., C.I.K., N.R.V., and L.J.J. reworked the manuscript. S.G. performed the genomic variant analysis of *A. oryzae* and produced Figure 4D. K.W., S.M., P.S., and S.G. offered feedback on the final manuscript, which J.D.E. and C.I.K. incorporated. A.E. provided funding for the experiment, metabarcoding, and metabolomics and advised on and hosted the experiment at the MIT Media Lab Space Exploration Initiative as part of M.C.’s project “Interplanetary Gastronomy.” J.D.E. provided funding for the metagenomics and genomics. All authors read and approved the manuscript before submission.

## Declaration of interests

The authors declare no competing interests.

## STAR★Methods

### Key resources table

**Table:** 

REAGENT or RESOURCE	SOURCE	IDENTIFIER
**Chemicals, peptides, and recombinant proteins**		

Sodium chloride (NaCl)	Sigma Aldrich	CAS: 7647-14-5
2-methyl-3-heptanone	Sigma Aldrich	CAS: 13019-20-0
Methanol	Sigma Aldrich	CAS: 67-56-1
Acetonitrile	Sigma Aldrich	CAS: 75-05-8

**Critical commercial assays**		

DNeasy PowerSoil Kit	Qiagen	ID: 47014

**Deposited data**		

Raw genomic data of *A. oryzae* strains	This paper	BioProject: PRJNA944135
Raw metagenomic data	This paper	BioProject: PRJNA944135
Raw metabolomic data and processed genomic and metagenomic data (BAM and VCF files, assembled contigs, predicted genes, marker genes, and genes encoding for enterotoxins)	This paper	Mendeley Data: https://data.mendeley.com/datasets/fspj4zzpjj/2

**Oligonucleotides**		

ITS1F & ITS4 primers	Op De Beeck et al.^98^	https://doi.org/10.1371/journal.pone.0097629

**Software and algorithms**		

fastp v.0.23.4	Chen et al.^99^	https://doi.org/10.1093/bioinformatics/bty560
MetaPhlAn v.3.0.4	Truong et al.^100^	https://doi.org/10.1038/nmeth.3589
megahit v.1.2.9	Li et al.^101^	https://doi.org/10.1093/bioinformatics/btv033
Prodigal v.2.6.3 & fetchMG, v.1.0	Ciccarelli et al.^102^ & Sunagawa et al.^103^	https://doi.org/10.1126/science.1123061 https://doi.org/10.1038/nmeth.2693
MaSuRCA 4.0.8	Zimin et al.^104^	https://doi.org/10.1093/bioinformatics/btt476
Augustus 3.4.0	Stanke and Morgenstern^105^	https://doi.org/10.1093/nar/gki458
FreeBayes 1.3.2	Garrison and Marth^106^	https://github.com/freebayes/freebayes
Thermo TraceFinder 4.1	ThermoFisher Scientific^107^	https://www.thermofisher.com/

### Experimental model and study participant details

A total of 14 panelists participated in the sensory analysis. All panelists (n = 14) tasted all miso samples (n = 3). None of the panelists had tasted the misos before. All panelists were adults, of which 6 were women, 7 men, and 1 non-binary. Beyond these panelists, 2 women and 1 man also participated in the sensory analysis, but their results were excluded from subsequent analysis because they had tasted the misos before (n = 2), or because they knew the assignment of codes to samples (n = 1). Personal data of panelists’ ancestry, race, ethnicity, and socioeconomic status were not deemed relevant to the study’s objectives, and so were not collected. The panelists were untrained, though all had some professional relation to food, flavor, and/or fermentation, for which they were selected. Panelists were identified and invited to participate through the authors’ networks around Copenhagen, Denmark.

The Technical University of Denmark, where the analysis was conducted, did not at the time of the study (and does not at the time of writing) have an Ethics Review Board or similar body, so ethics approval was not possible to obtain through institutional channels. Instead, as advised by central university administrators on research ethics, we prepared an ethics and data management plan based on previous studies conducted by the lead contact that had obtained ethics approval, and in accordance with EU GDPR regulation. All panelists granted their informed consent to participate, and for their data (personal and experimental) to be collected, stored, anonymized, analyzed, and published in accordance with our ethics and data management plan.

### Method details

#### Experimental design

The miso mixture was prepared using cooked soybeans (Organic Soybeans from Rømer A/S, Denmark), rice kōji (rice fermented with the filamentous fungus Aspergillus oryzae; Italian-grown sushi rice from Grøntgrossisten A/S, Denmark; albino rice kōji spores from Bio’c, Japan), and salt (Fine Atlantic Sea Salt from Urtekram A/S, Denmark). A high-kōji low-salt style of young miso (soybean:kōji ratio of 1:1 and 4 % salt w/w) was selected to facilitate a faster fermentation appropriate for the 30-day period. The miso mixture was produced in Copenhagen, Denmark, in a food-safe environment according to a HACCP plan^32^^,^^35^ split in two portions (one third and two thirds), sealed in plastic vacuum bags and frozen. One third was kept in Copenhagen and two thirds were shipped frozen to Cambridge, Massachusetts, USA. Before launch, the miso mixture was thawed and packed into identical cylindrical clear polystyrene containers (diameter = 6.35 cm, height = 5.46 cm, volume = 177 ml) under flow hood, with the lid semi-sealed to allow off-gassing. One was kept in Copenhagen (KBH), one in Cambridge (CAM), and the third was transported to the International Space Station (ISS). ‘KBH’ comes from the word for Copenhagen in Danish, ‘København’, to differentiate it more from ‘CAM’ than ‘CPH’ would.

#### Environmental data measurements

While fermenting, the ISS and CAM misos were contained in environmental sensing boxes, which measured temperature, humidity, pressure, off-gassing, light and radiation. The radiation sensor we used (MightyOhm Geiger Counter) generates a signal, also known as a ‘click’, whenever beta or gamma radiation collides with the gas (a mixture of neon, bromine, and argon) inside a sealed tube. We recorded each collision over the course of the experiment and calculated an hourly rate.

Power was interrupted a few times on the ISS over the course of the experiment, which meant that the environmental data had some gaps. See Figure S1 in Document S1 for a graph showing the periods of interruption for temperature, and Table S1 for the raw environmental data. More information about the environmental sensing box, its design and engineering can be found in Coblentz et al.^108^

Temperature and humidity for the Copenhagen miso were measured manually using a basic thermometer (Silvan A/S, Denmark) and hygrometer (TFA, Germany).

#### Sample preparation

Samples of the starting miso mixture were not taken, as our main question was about how the final misos were similar to and/or different from each other, rather than about how the fermentation process shaped the microbiota over time, which other studies have investigated.^32^^,^^35^ So this baseline was not needed.

After the fermentation the misos were separated into three portions—top, middle, and bottom—to investigate how the microbial communities and flavour compounds might differ within the sample. The top portion was the surface of the miso where there was fungal growth, tamari (a soy-sauce like liquid that often collects on top of a miso as it ferments), and oxidized miso paste. This portion is usually removed before consumption. From the level at which there was no longer oxidation, this remainder was split in two, yielding the middle and bottom portions.

#### DNA sequencing

##### Shotgun metagenomics

For shotgun metagenomic analysis, DNA was extracted using 1 g of each miso portion (top, middle and bottom). The samples were diluted in 9 ml of saline water (0.9 % NaCl), placed in stomacher bags (BagPage, Interscience, France) and homogenized in a laboratory blender Stomacher 400 (Seward Co.) at high speed for 2 min. This mixture was filtered (filter porosity of 280 microns) and subsequently centrifuged to concentrate the cells. The pellet was then used for DNA extraction using the Qiagen DNeasy PowerSoil Kit. The metagenomics DNA libraries were prepared using plexWell kit and the library pool was sequenced on an Illumina Nextseq 500 instrument using Nextseq Midoutput 300-cycle kit v.2.5 at the Novo Nordisk Foundation Center for Biosustainability, Technical University of Denmark (Lyngby, Denmark).

Quality control and preprocessing of fastq files were performed with fastp.^99^ First we analyzed the samples separated by top, middle and bottom portions for each miso, and then we assembled the reads of the portions belonging to the miso from each location. From the trimmed fastq files, we estimated microbial composition by mapping the sample reads against the representative catalog contained in the MetaPhlAn tool, v.3.0.4^100^ using default parameters and --read_min_len 70. Paired-end reads were assembled into contigs using megahit 1.2.9,^101^ with default settings. We then predicted genes using Prodigal (v.2.6.3) and marker genes were extracted using fetchMG, v.1.0.^102^^,^^103^ Thereafter, taxonomic assignments were made using the leuS marker gene, whose closest homologue was assigned by a BLAST search on all available sequences in the NCBI protein database. Species composition plots were created in R (v.3.6.1) using the package ggplot2, v.3.3.2.

Since some Staphylococcus species were found in the samples, a search was conducted for any genes encoding for enterotoxins. Genes encoding for PTSAgs (sea-see, seg-sevu, selv, selx, sey, selz, sel26, sel27 and TSST1) and exfoliative toxins (eta, etb, etd) were searched for by BLASTing these genes against the assembled metagenomes. Following Zhou et al., 2021,^109^ reliable virulence genes were confirmed if sequence identity was > 80 % and query coverage was > 80 %.

##### Whole genome sequencing of *A. oryzae*

This analysis was conducted to investigate the mutation rate. For analysis of SNPs it is more reliable to sequence the whole genomes of isolated strains rather than reconstructing genomes from the metagenomic data, because in the recovery of metagenome-assembled genomes (MAGs) genes can be lost. *A. oryzae* was selected for this purpose, because it was known to be in each miso and that it would grow on plates. *A. oryzae* strains were isolated from the kōji used for the miso preparations (for our reference strain), from all portions of the ISS miso, and from the bottom of all the misos (ISS, CAM and KBH). The bottom was selected because this is where the highest abundance of *A. oryzae* was detected in the metagenomics data. 1 g of each sample was homogenized with saline solution (0.9 % NaCl) and plated in Potato Dextrose Agar (PDA) with ampicillin (0.05 g per 500 ml of media) and chloramphenicol (0.02 g per 500 ml of media) to inhibit bacterial growth. Then, the plates were incubated at 30°C for 5 days. The strains that grew were cut in small pieces, placed in stomacher bags (BagPage, Interscience, France) with 5 ml of saline water and homogenized in a laboratory blender Stomacher 400 (Seward Co.) at high speed for 2 min. The mixture was centrifuged and the pellet was used for DNA extraction using Qiagen DNeasy PowerSoil Kit. The DNA concentration and quality was evaluated using NanoDrop ND-1000 spectrophotometer (NanoDrop Technology Inc., Wilmington, DE, USA) and with Qubit 2.0 fluorometer (Life Technologies) using a Qubit dsDNA HS (High Sensitivity) Assay Kit. The preliminary species assignation was performed by sequencing the ITS region, using ITS1F (CTTGGTCATTTAGAGGAAGTAA) and ITS4 (TCCTCCGCTTATTGATATGC).^98^

The genomic DNA was sent to Beijing Genome Institute (BGI, Wuhan, China) for constructing a short insert library and sequenced to produce paired-end reads of 150 bp on a DNBSEQ-G400 platform. For each strain, the fastq sequences were assembled with MaSuRCA 4.0.8^104^ and annotations were performed using Augustus 3.4.0.^105^

For the Single Nucleotide Variants (SNPs) analysis, first, the paired fastq reads of each sample were mapped using bowtie2 v.2.4.2 to the fasta of the reference genome of *A. oryzae* RIB40. The SAM file outputs were converted to BAM files and sorted using samtools 1.14. FreeBayes 1.3.2 was run with --ploidy 1 (https://github.com/freebayes/freebayes) and vcf output was filtered with “VCFfilter”, QUAL>20.^106^ All missing genotypes were filtered out, as well as mutations between strain RIB40 and our reference strain, to analyze only the variations occurring during fermentation. Next, vcfstats was applied and the number of variants (SNPS, MNPs and indels) of *A. oryzae* from KBH, CAM and ISS miso in relation to the reference strain was calculated, and their placement in the genome was analyzed.

#### Metabolomics

##### Volatile aroma compounds

For each sample, 5 g of miso was homogenized with 2.2 times of distilled water, vortexed, shaken in a test tube at 25°C for 2 h, filtered and 4 ml transferred to 10 ml airtight vials. To each vial, 1.44 g NaCl (Sigma Aldrich, St. Louis, MO, USA) was added. Ten microliters of 2-methyl-3-heptanone (diluted to 27.2 mg/l in LC/MS-grade methanol) was added as an internal standard. Solid-phase microextraction (SPME) fiber coated with 65 μm polydimethylsiloxane/divinylbenzene (PDMS/DVB) was used for the extraction of volatile compounds. The GC/MS analysis was performed on a Thermo Scientific TRACE 1310 Gas Chromatograph equipped with a Thermo Scientific Q Exactive Orbitrap mass spectrometry system using a Thermo fused-silica capillary column of cross-linked TG-5SILMS GC columns (30 m x 0.25 mm x 0.25 μm; ThermoFisher Scientific, Waltham, MA, USA).^110^ Identification was performed by comparison of the MS spectra with the NIST library and with the Retention Index (Kovat indices). Concentration values were expressed as 2-methyl-3-heptanone equivalent (μg/l). Data were acquired and analyzed with Thermo TraceFinder 4.1 software package (ThermoFisher Scientific, Waltham, MA, USA).^107^

##### Free amino and organic acids

800 mg of each sample was prepared by adding 5 ml of methanol (80 % v/v; EMD OmniSolv® LCMS Methanol, Sigma Aldrich, St. Louis, MO, USA) and 0.1 μl of internal standard (IS), a mix of 17 amino acids. Samples were vortexed for two minutes, centrifuged at 1000 rpm for 20 min, and the supernatants were transferred to microcentrifuge tubes. Then, samples were dried under N2 flow and resuspended in 100 μl of acetonitrile 30 % (Sigma Aldrich, St. Louis, MO, USA) in ultrapure water (Arium Pro Ultrapure Water System, Sartorius, Goettingen, Germany). Standards were prepared in a solution of methanol (80 % v/v; EMD OmniSolv® LCMS Methanol, Sigma Aldrich, St. Louis, MO, USA) and IS with the following concentrations: all amino acids 40 μM, except asparagine (Asn) and glutamine (Gln) at 100 μM, and all organic acids at 100 μM. 1 ml of each standard was dried down and resuspended similarly to the samples. All samples were run on a Vaquish HPLC and Orbitrap™ IQ-X™ Tribrid™ Mass Spectrometer (ThermoFisher Scientific, Waltham, MA, USA). The LC conditions were the following: the column maintained at 40°C (Hilicon iHILIC column 150x2.1 mm 5 micron, Agilent Technology, Santa Clara, CA, US). The mobile phase A consisted of 20 mM ammonium carbonate, 0.1 % ammonium hydroxide, in water; phase B was composed acetonitrile 97 %, in water, and flow rate from 0.05 to 0.15 (ml/min) for 45 min. LC was connected to an Orbitrap ID-Z tribid by heated electrospray ionization (±HESI). The MS parameters were as follows: mz range: 65 to 1000. Internal calibration used. Data were acquired and analyzed with Thermo TraceFinder 4.1 software package (ThermoFisher Scientific, Waltham, MA, USA).^107^

#### Sensory analysis

All three misos were tasted: ISS, KBH, and CAM. The top portion was removed and the middle and bottom portions were mixed together, as miso is eaten traditionally. Each sample (5 g) was served as a paste, double-blind, in a transparent, plastic container, with a lid to keep the aromas in, and coded with a different randomly generated 3-digit number. The samples were served to the panelists at room temperature in a room with controlled temperature and relative humidity (18±2°C; 74 ± 5% RH); the illumination was a combination of natural and nonnatural light. The order of evaluation was randomized. All the samples were served at the same time and panelists tasted them from left to right. It was mandatory for the panelists to rinse their mouths with water between samples. The protection and processing of personal data was followed by the Declaration of Helsinki and the 2016/679 EU Regulation.

Panelists were invited to answer preliminary questions such as ‘What is your main profession?’, ‘Have you tried miso before?’ and ‘If so, how often on average do you eat it?’ using a 5-point scale, to collect more information about their background, experience and habits (Table S4.1). The session was conducted with three different tests: CATA (Check-All-That-Apply), Hedonic Rating, and Projective Mapping. CATA is a structured question format in which respondents are presented a list of terms and asked to select all those that apply to the sample.^111^ Hedonic rating asks respondents about their liking of the sample, often using a numbered point scale, which can be used to assess enjoyment and acceptance.^112^ In Projective Mapping (PM), respondents are asked to position samples on a sheet of paper according to their similarity (closer together) and difference (further apart).^113^

CATA was conducted with a total of 31 attributes chosen based on the volatile compound analysis and including the basic tastes (Table S4.2). Panelists had the option to write other attributes perceived. Sixteen extra attributes were gathered, for a total of 47 (Table S4.2). Duplicated and closely-related low-scoring attributes were consolidated for clarity,^114^^,^^115^^,^^116^^,^^117^ yielding 36 distinct attributes for the final analysis (Table S4.2). For liking, panelists rated the samples using a 9-point hedonic scale (1=extremely dislike, 9= extremely like; see Table S4.3 for the raw CATA and liking data). Lastly, the Projective Mapping test was conducted to evaluate how similar or different the panelists considered the samples (Table S4.6).

For the same samples served in the tasting, a colorimeter was used to determine the color of the misos (FRU WR10Q, Longgang, Shenzhen, China) using the CIELAB color coordinates L∗ (lightness), a∗ (red-green), and b∗ (blue-yellow; Table S4.7). Though not a sensory analysis test, we report this data alongside the sensory analysis data to support it.

### Quantification and statistical analysis

#### Environmental data measurements

Mean and standard deviation (SD) were first calculated for each of the sensors individually. Values for each environmental parameter were subsequently reported as the average of the individual sensor means if there was no statistical difference (ANOVA, p>0.05) between the two sensors; otherwise the individual values were reported separately for sensors 1 and 2. The average of the SDs was calculated based on the formula

$$\text{Average}\;\text{SD}=\sqrt{\left({s_{1}}^{2}+{s_{2}}^{2}+...+{s_{k}}^{2}\right)\;/\;k\;}$$

where s_k_ is the standard deviation for the k^th^ group, and k is the total number of groups.

The precision of mean value for each of the environmental parameters was reported to a uniform number of decimal places, based on the least precise SD value amongst the three sites when taken to 2 significant figures.

A standard deviation for radiation was not calculated because the interruption periods were non-uniform so it would not be representative.

#### Metabolomics data analysis

All data (raw and processed) from metabolomics analysis are reported to 2 decimal places in Table S3 (mean and SD columns are highlighted in grey). Average volatile aroma compound concentration (Figure 5A) was calculated by taking the mean of each compound class across the three positional fractions within each miso. SD was calculated based on the total mean concentration of all volatile aroma compounds in each miso. Mean and SD values were reported in the main text to a uniform number of decimal places, based on the least precise SD value among the three sites when taken to 2 significant figures. Comparative fold changes between samples were calculated based on the calculated means before rounding.

#### Sensory analysis

A one-way ANOVA of liking was conducted to investigate if there was significant difference in liking between samples (Table S4.4). Tukey’s HSD was used as a post-hoc test. Two-way ANOVA and Tukey’s multiple comparisons test were used to compare liking according to gender (Table S4.4). No significant differences according to gender were found. CATA data were analyzed using Cochran's Q with pairwise comparisons based on the McNemar-Bonferroni approach to identify significant differences among attributes.^111^ These values vary on a scale from 0 to 1 (Table S4.5), based on the raw dichotomic data (Table S4.3). Statistical analyses were conducted using XLSTAT Version 2009.6.03.^118^ Results were considered significant when p < 0.05.

Projective Mapping was conducted using projective mapping data analysis (Table S4.6). The Projective Mapping method is visualized in Figure S2 (Document S1). Based on these data, overall sensory difference between samples was quantified by the following method:

1. Calculate the distance (D) between each pair of samples for each panelist:
  $$D_{ISS-KBH}=\sqrt{\;|{\left(X_{ISS}-X_{KBH}\right)|}^{2}+|{\left(Y_{ISS}-Y_{KBH}\right)|}^{2}}$$
  $$D_{ISS-CAM}=\sqrt{\;|{\left(X_{ISS}-X_{CAM}\right)|}^{2}+|{\left(Y_{ISS}-Y_{CAM}\right)|}^{2}}$$
  $$D_{KBH-CAM}=\sqrt{\;|{\left(X_{KBH}-X_{CAM}\right)|}^{2}+|{\left(Y_{KBH}-Y_{CAM}\right)|}^{2}}$$
  which establishes each panelist’s perceived difference between each pair of samples.
2. Calculate the mean (M) of distances for each sample pair across all panelists:
  $$M_{ISS-KBH}==\frac{D_{ISS-KBH\left(1\right)}+D_{ISS-KBH\left(2\right)}+D_{ISS-KBH\left(3\right)}+\ldots +D_{ISS-KBH\left(n\right)}}{n}$$
  $$M_{ISS-CAM}==\frac{D_{ISS-CAM\left(1\right)}+D_{ISS-CAM\left(2\right)}+D_{ISS-CAM\left(3\right)}+\ldots +D_{ISS-CAM\left(n\right)}}{n}$$
  $$M_{KBH-CAM}==\frac{D_{KBH-CAM\left(1\right)}+D_{KBH-CAM\left(2\right)}+D_{KBH-CAM\left(3\right)}+\ldots +D_{KBH-CAM\left(n\right)}}{n}$$
  which establishes the average difference perceived for each pair of samples across all panelists (where n is the number of panelists; here n = 14).
3. Calculate the factor (F) by which ISS differs from KBH and from CAM:
  $$F_{ISS-KBH}=\frac{M_{ISS-KBH}}{M_{KBH-CAM}}$$
  $$F_{ISS-CAM}=\frac{M_{ISS-CAM}}{M_{KBH-CAM}}$$
  which establishes by how much more the space miso was perceived as different from KBH and from CAM, compared to the perceived difference between the two earth misos.

For full calculations see Table S4.6.

## Supplemental information

Document S1. Figures S1 and S2

Table S1. Environmental data, related to Figure 3 Environmental data for the misos including temperature, humidity, pressure, and radiation, as well as spatiotemporal tracking of the three samples.

Table S2. Metagenomics, related to Figure 4 Metagenomics data from the misos mapped to the MetaPhlAn database, *leuS* marker gene analysis of the metagenomic data, genomic analysis of *A. oryzae* from the three misos, and toxin analysis of the misos based on the metagenomics data mapped to NCBI BLAST.

Table S3. Metabolomics, related to Figures 5 and 6 Metabolomics data for the three misos, of volatile compounds, free amino acids, and organic acids, with statistical analysis.

Table S4. Sensory analysis, related to Figure 7 Sensory analysis data, including the questionnaire, attributes, liking and CATA data, ANOVA, Tukey's test, Cochran’s Q test, Projective Mapping, and colorimetry.
