# Supplementary material for: Food fermentation in space: Opportunities and challenges
Source: iScience. 2025 Apr 2;28(4):112189. doi: 10.1016/j.isci.2025.112189 (PMC12131251; doi:10.1016/j.isci.2025.112189)
Supplement: Document S1. Figures S1 and S2 [file mmc1.pdf]

iScience, Volume *28*

Supplemental information

Food fermentation in space:

Opportunities and challenges

Maggie Coblentz, Joshua D. Evans, Caroline Isabel Kothe, Tiffany Mak, Nabila Rodríguez Valerón, Patrick Chwalek, Kim Wejendorp, Shilpa Garg, Louisa Pless, Sarah Mak, Pia M. Sörensen, Leonie Johanna Jahn, and Ariel Ekblaw

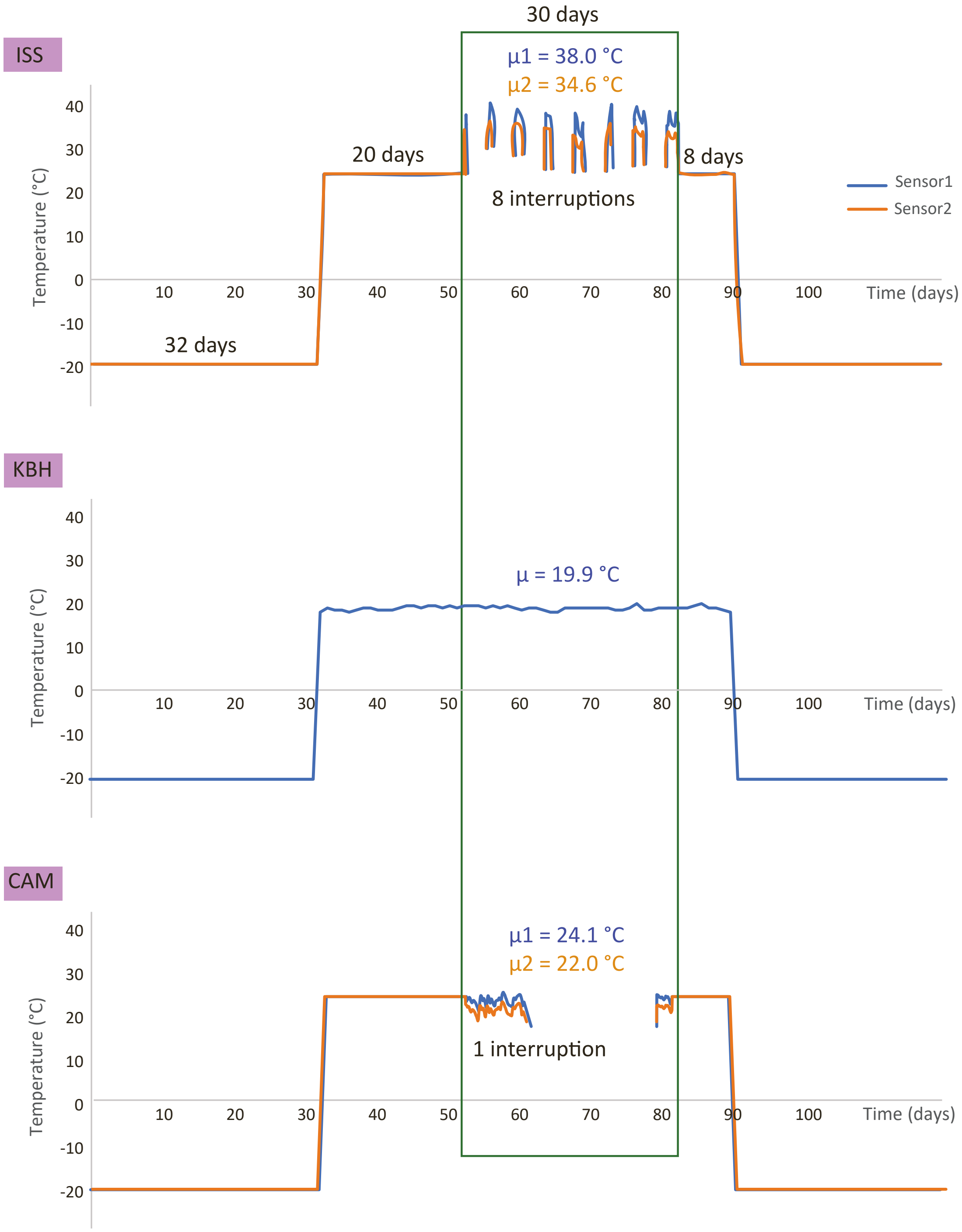

Figure S1. Temperature data for ISS, KBH, and CAM misos, with interruptions. Related to STAR Methods.

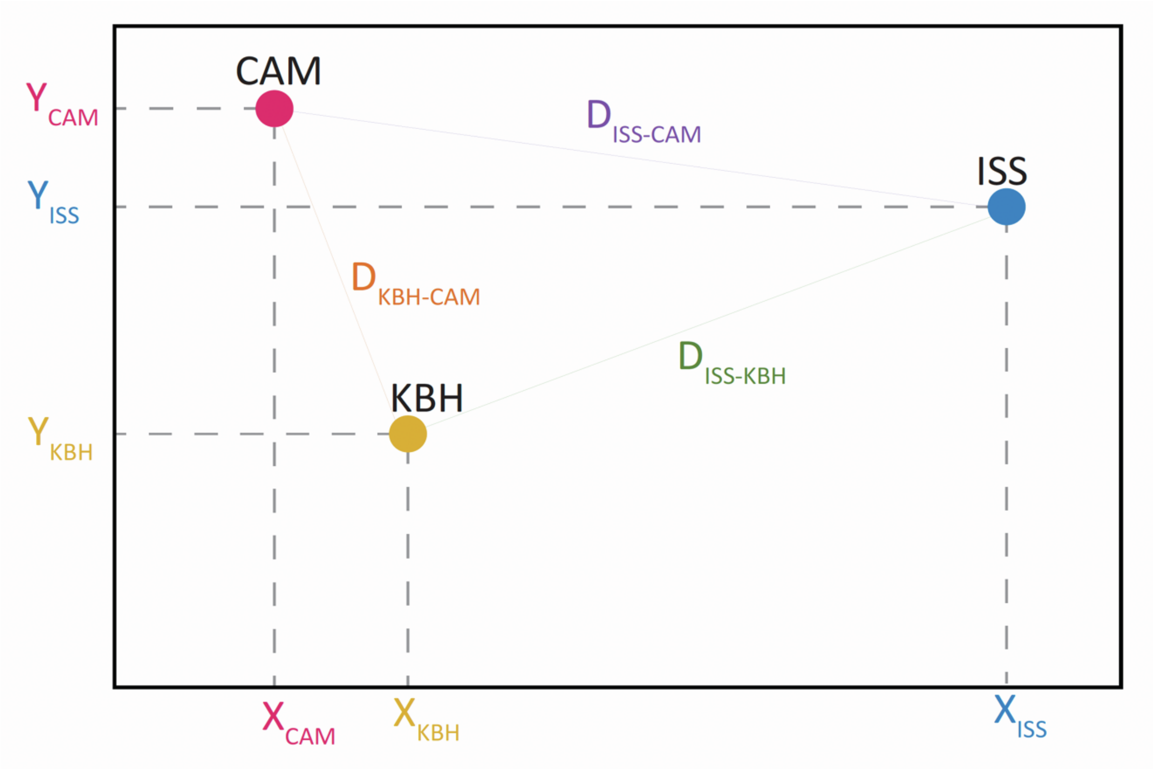

Figure S2. An example of the projective mapping of the samples to help visualize how the overall sensory difference was calculated. Related to STAR Methods.

Supplementary References

S1. Zhou, M., Wu, Y., Kudinha, T., Jia, P., Wang, L., Xu, Y., and Yang, Q. (2021). Comprehensive Pathogen Identification, Antibiotic Resistance, and Virulence Genes Prediction Directly From Simulated Blood Samples and Positive Blood Cultures by Nanopore Metagenomic Sequencing. Front Genet *12*, 1–11. https://doi.org/10.4014/mbl.1902.02007.

S2. Ding, W., Liu, Y., Zhao, X., Peng, C., Ye, X., Che, Z., Liu, Y., Liu, P., Lin, H., Huang, J., et al. (2021). Characterization of volatile compounds of Pixian Douban fermented in closed system of gradient steady-state temperature field. Food Sci Nutr *9*, 2862–2876. https://doi.org/10.1002/fsn3.2242.

S3. Dulce, V.R., Anne, G., Manuel, K., Carlos, A.A., Jacobo, R.C., Sergio de Jesús, C.E., and Eugenia, L.C. (2021). Cocoa bean turning as a method for redirecting the aroma compound profile in artisanal cocoa fermentation. Heliyon *7*. https://doi.org/10.1016/j.heliyon.2021.e07694.

S4. Rapp, C., Pival-Marko, S., Tassano, E., Nidetzky, B., and Kratzer, R. (2021). Reductive enzymatic dynamic kinetic resolution affording 115 g/L (S)-2-phenylpropanol. BMC Biotechnol *21*. https://doi.org/10.1186/s12896-021-00715-5.

S5. Li, J., Peng, B., Huang, L., Zhong, B., Yu, C., Hu, X., Wang, W., and Tu, Z. (2023). Association between flavors and microbial communities of traditional Aspergillus-Douchi produced by a typical industrial-scale factory. Lwt *176*, 114532. https://doi.org/10.1016/j.lwt.2023.114532.

S6. Wang, X., Fan, W., and Xu, Y. (2014). Comparison on aroma compounds in Chinese soy sauce and strong aroma type liquors by gas chromatography–olfactometry, chemical quantitative and odor activity values analysis. European Food Research and Technology *239*, 813–825. https://doi.org/10.1007/s00217-014-2275-z.

S7. Zhao, J., Dai, X., Liu, X., Zhang, H., Tang, J., and Chen, W. (2011). Comparison of aroma compounds in naturally fermented and inoculated Chinese soybean pastes by GC-MS and GC-Olfactometry analysis. Food Control *22*, 1008–1013. https://doi.org/10.1016/j.foodcont.2010.11.023.

S8. Ogunremi, O.R., Agrawal, R., and Sanni, A. (2020). Production and characterization of volatile compounds and phytase from potentially probiotic yeasts isolated from traditional fermented cereal foods in Nigeria. Journal of Genetic Engineering and Biotechnology *18*, 16–23. https://doi.org/10.1186/s43141-020-00031-z.

S9. Yang, H., Wu, D., Guo, D., and Lu, J. (2019). The aromatic volatile composition of Lonicera edulis wines produced with three different strains of Saccharomyces cerevisiae. Journal of the Institute of Brewing *125*, 100–109. https://doi.org/10.1002/jib.542.

S10. Nan, L., Liu, L., Li, Y., Huang, J., Wang, Y., Wang, C., Wang, Z., and Xu, C. (2021). Comparison of Aroma Compounds in Cabernet Sauvignon Red Wines from Five Growing Regions in Xinjiang in China. J Food Qual *2021*. https://doi.org/10.1155/2021/5562518.

S11. Kumazawa, K., Kaneko, S., and Nishimura, O. (2013). Identification and characterization of volatile components causing the characteristic flavor in miso (Japanese fermented soybean paste) and heat-processed miso products. J Agric Food Chem *61*, 11968–11973. https://doi.org/10.1021/jf404082a.

S12. Meng, Q., Imamura, M., Katayama, H., Obata, A., and Sugawara, E. (2017). Key compounds contributing to the fruity aroma characterization in Japanese raw soy sauce.

Biosci Biotechnol Biochem *81*, 1984–1989. https://doi.org/10.1080/09168451.2017.1364620.

S13. Kłosowski, G., Mikulski, D., and Pielech-Przybylska, K. (2021). Pyrazines biosynthesis by bacillus strains isolated from natto fermented soybean. Biomolecules *11*, 1–12. https://doi.org/10.3390/biom11111736.

S14. Yan, Y., Chen, S., Nie, Y., and Xu, Y. (2021). Quantitative analysis of pyrazines and their perceptual interactions in Soy Sauce aroma type Baijiu. Foods *10*, 1–12. https://doi.org/10.3390/foods10020441.

S15. Gao, X., Feng, T., Sheng, M., Wang, B., Wang, Z., Shan, P., Zhang, Y., and Ma, H. (2021). Characterization of the aroma-active compounds in black soybean sauce, a distinctive soy sauce. Food Chem *364*, 130334. https://doi.org/10.1016/j.foodchem.2021.130334.

S16. Kim, M.K., Chung, H.J., and Bang, W.S. (2018). Correlating physiochemical quality characteristics to consumer hedonic perception of traditional Doenjang (fermented soybean paste) in Korea. J Sens Stud *33*, 1–10. https://doi.org/10.1111/joss.12462.

S17. Cherniienko, A., Pawełczyk, A., and Zaprutko, L. (2022). Antimicrobial and Odour Qualities of Alkylpyrazines Occurring in Chocolate and Cocoa Products. Applied Sciences (Switzerland) *12*. https://doi.org/10.3390/app122211361.

S18. Sun, J., Li, Q., Luo, S., Zhang, J., Huang, M., Chen, F., Zheng, F., Sun, X., and Li, H. (2018). Characterization of key aroma compounds in Meilanchun sesame flavor style baijiu by application of aroma extract dilution analysis, quantitative measurements, aroma recombination, and omission/addition experiments. RSC Adv *8*, 23757–23767. https://doi.org/10.1039/c8ra02727g.
